# Molecular insights into the interplay between type 2 diabetes mellitus and osteoporosis: implications for endocrine health

**DOI:** 10.3389/fendo.2024.1483512

**Published:** 2025-01-17

**Authors:** Liyun Jiang, Xia Song, Li Yan, Yali Liu, Xiumei Qiao, Wen Zhang

**Affiliations:** ^1^ Medical Laboratory Center, The First Hospital of Lanzhou University, Lanzhou, China; ^2^ Medical Laboratory Center, Gansu Provincial People’s Hospital, Lanzhou, China

**Keywords:** type 2 diabetes mellitus, osteoporosis, bone metabolism, autophagy, ferroptosis, perirenal fat thickness, vitamin D receptor gene polymorphism

## Abstract

**Background:**

Type 2 diabetes mellitus (T2DM) and osteoporosis are prevalent, interconnected chronic diseases that significantly impact global health. Understanding their complex biological relationship is crucial for improving patient outcomes and treatment strategies.

**Method:**

This review examines recent research on the mechanisms linking T2DM with osteoporosis. It focuses on how abnormalities in bone metabolism, autophagy, ferroptosis, and vitamin D receptor (VDR) gene polymorphisms contribute to osteoporosis in T2DM patients.

**Results:**

Our analysis indicates that T2DM is associated with reduced bone formation and increased bone resorption, which are influenced by hormonal changes, inflammation, and disrupted cellular signaling pathways. Additionally, increased perirenal fat thickness worsens osteoporosis through local inflammation and altered adipokine levels. VDR gene polymorphisms provide new molecular insights into this connection.

**Conclusion:**

Addressing the identified mechanisms with targeted management strategies may improve bone health in individuals with T2DM. Future research should explore these associations in greater detail to develop more effective prevention and treatment strategies.

## 1 Introduction

Type 2 diabetes mellitus (T2DM) is a common chronic endocrine disorder affecting millions of people worldwide, with its prevalence continuously rising globally, making it a significant public health concern that poses a serious threat to human health (1). T2DM not only leads to abnormal blood sugar regulation but is also associated with various complications, including cardiovascular diseases, kidney disease, retinopathy, and neuropathy (2). In recent years, more research has shown a complex biological connection between T2DM and osteoporosis, with T2DM patients having a significantly higher incidence of osteoporosis than non-diabetic individuals (3). Osteoporosis is a disease characterized by reduced bone density and structural deterioration of bone tissue, significantly increasing the risk of fractures and severely impacting the quality of life of patients (4). Therefore, exploring the mechanisms linking T2DM and osteoporosis holds significant clinical significance and societal value.

**Figure 1:**
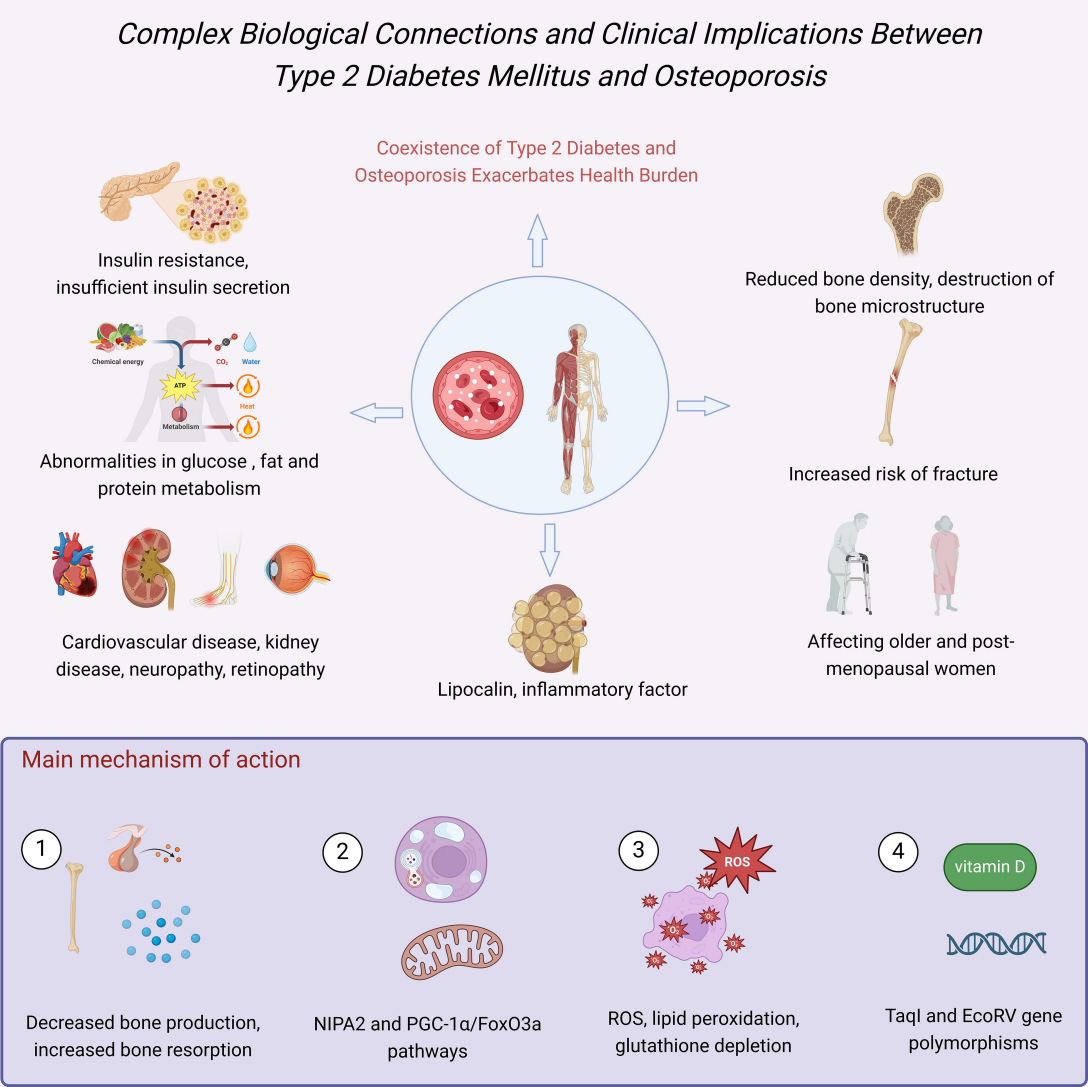
Complex biological connections and clinical implications between T2DM and osteoporosis.

In recent years, domestic and foreign scholars have conducted extensive research on the mechanisms linking T2DM and osteoporosis, identifying multiple factors that may play a role in this association (5) (**Figure 1**). Among them, abnormal bone metabolism, autophagy, ferroptosis, and vitamin D receptor (VDR) gene polymorphism have garnered much attention for their impact on bone health (6). Studies have indicated that in patients with T2DM, decreased bone formation and increased bone resorption are associated with various factors, including changes in hormone levels, increased inflammatory status, and abnormalities in cell signaling pathways (7). Additionally, an increase in perirenal fat thickness may exacerbate osteoporosis by affecting local inflammation and levels of adipokines (8). VDR gene polymorphisms have also been found to be associated with the development of diabetes-related osteoporosis (9). In conclusion, there exists a complex biological connection between T2DM and osteoporosis, which not only increases the health burden on patients but also poses new challenges for clinical interventions and treatment strategies.

Specifically, the decreased bone marrow mesenchymal stem cell (BMSC) osteogenic efficiency in patients with T2DM is considered one of the fundamental mechanisms leading to osteoporosis (10). Through RNA sequencing technology and bioinformatic analysis, researchers have found that differential gene expression in BMSCs of T2DM patients significantly reduces their osteogenic capability (11). For instance, the downregulation of FOXQ1 is closely associated with developing osteoporosis in T2DM (12). Additionally, ferroptosis involves cell death related to oxidative stress and iron homeostasis. Studies suggest that elevated blood glucose levels may induce ferroptosis in bone cells by enhancing reactive oxygen species (ROS), lipid peroxidation, and glutathione depletion (13). Mitochondrial ferritin protects bone cells from ferroptosis by reducing oxidative stress caused by excess ferrous ions, thus playing a protective role in bone health (14). Furthermore, autophagy, especially mitophagy, is crucial in maintaining cellular physiological function and health (15). Research indicates that NIPA2 and the PGC-1α/FoxO3a pathway play critical regulatory roles in mitophagy in T2DM-related osteoporosis (16).

Increased perirenal fat thickness may affect the bone health of patients with T2DM through multiple mechanisms (17). Studies have shown that perirenal fat exhibits high energy metabolism and adipokine secretion activity, and these adipokines directly influence bone remodeling and reconstruction (18). For example, leptin is highly active in perirenal fat and may disrupt bone formation and resorption balance by generating inflammatory factors (19). Additionally, VDR gene polymorphisms are closely associated with T2DM-related osteoporosis (20). Research has indicated that TaqI and EcoRV gene polymorphisms are linked to increased susceptibility to osteoporosis in female patients with T2DM (21). In conclusion, there is a clear biological association between T2DM and osteoporosis, and the coexistence of these diseases exacerbates the health burden on patients. Comprehensive management strategies and treatments targeting these mechanisms may help improve the bone health of these patients (22). Future research should further explore the details of these associated mechanisms to develop more effective prevention and treatment strategies.

## 2 Basic knowledge of T2DM and osteoporosis

### 2.1 Introduction to T2DM

T2DM is a complex metabolic disease characterized by insulin resistance and inadequate insulin secretion, leading to sustained high blood sugar levels. In recent years, with lifestyle changes and rising obesity rates, the prevalence of T2DM has significantly increased, becoming a global epidemic (1). T2DM affects glucose metabolism and is closely related to abnormalities in fat metabolism and protein metabolism, thereby causing various complications (23). These complications include cardiovascular diseases, kidney diseases, neuropathy, retinopathy, etc., severely impacting the quality of life and life expectancy of patients (24). Additionally, T2DM can lead to abnormalities in bone metabolism, increasing the risk of osteoporosis and further burdening patients’ health (4).

### 2.2 Introduction to osteoporosis

Osteoporosis is a bone disease characterized by reduced bone density and structural deterioration, leading to an increased risk of fractures (25). Osteoporosis is often referred to as the “silent disease” because patients may not have noticeable symptoms before experiencing fractures (5). The condition primarily affects the elderly population, especially postmenopausal women, but can also occur in men and individuals of other age groups (8). The development of osteoporosis is complex and involves various factors such as genetic factors, hormonal changes, nutritional status, and lifestyle (3). An imbalance in bone metabolism is a crucial characteristic of osteoporosis, where bone resorption exceeds bone formation, leading to decreased bone mass and strength (6).

### 2.3 The association between T2DM and osteoporosis

In patients with T2DM, the incidence of osteoporosis is significantly higher than in the general population, mainly due to the metabolic disturbances and the impact of complications caused by diabetes on bone metabolism (9). Under high blood sugar conditions, oxidative stress and inflammatory responses are enhanced, leading to damage and functional impairment of bone cells (10). Insulin resistance affects blood sugar control and directly influences bone density by inhibiting bone formation and promoting bone resorption (12). Additionally, T2DM patients often have vitamin D deficiency, increasing the risk of osteoporosis, as vitamin D plays a crucial role in calcium absorption and bone health (26). Studies have also found that the differentiation potential of BMSCs in T2DM patients decreases, significantly reducing bone formation capacity (2).

The basic mechanisms of osteoporosis involve multiple complex biological processes. Firstly, high blood sugar and insulin resistance caused by T2DM affect bone metabolism through various mechanisms, increasing the risk of osteoporosis (1). Secondly, the metabolic disturbances and complications of T2DM further exacerbate the progression of osteoporosis (24). For example, under high blood sugar conditions, enhanced oxidative stress and inflammatory responses lead to bone cell damage, while insulin resistance directly affects bone density by inhibiting bone formation and promoting bone resorption (25). Additionally, vitamin D deficiency and dysfunction of BMSCs also play essential roles in the osteoporosis of T2DM patients (4). Understanding these foundational mechanisms is crucial for further investigating the relationship between T2DM and osteoporosis, aiding in developing new prevention and treatment strategies to improve patients’ quality of life (5).

## 3 Decreased bone formation efficiency of BMSCs

BMSCs play a crucial role in the generation and repair of bone tissue. BMSCs have multipotent differentiation potential and can differentiate into various cell types, such as osteoblasts, chondrocytes, and adipocytes. BMSCs maintain bone homeostasis and function in healthy individuals by promoting bone formation. However, in patients with T2DM, the osteogenic differentiation ability of BMSCs significantly decreases, leading to reduced bone formation efficiency. This change impacts the bone’s quality and strength and increases the risk of osteoporosis (1). Studies have shown that the high blood sugar environment and insulin resistance in T2DM patients are the main reasons for the functional impairment of BMSCs (2).

High blood sugar is a significant characteristic of T2DM, and its impact on BMSCs has been confirmed in multiple studies. Under high blood sugar conditions, the proliferation capacity and osteogenic differentiation potential of BMSCs are significantly inhibited (4). The mechanism behind this phenomenon may involve several aspects, including oxidative stress, inflammatory responses, and accumulation of advanced glycation end products (AGEs). Oxidative stress is a common metabolic disorder in high blood sugar environments, increasing the levels of ROS within cells and leading to cellular dysfunction and DNA damage (5). Additionally, high blood sugar can activate inflammatory pathways such as nuclear factor kappa B (NF-κB), causing an increase in the secretion of inflammatory cytokines such as tumor necrosis factor-alpha (TNF-α) and interleukin-6 (IL-6), further inhibiting the osteogenic differentiation of BMSCs (3). Furthermore, the accumulation of AGEs not only alters the structure and function of the extracellular matrix but also, through interaction with their receptor (RAGE), activates multiple signaling pathways that inhibit the osteogenic capacity of BMSCs (25).

In addition to the direct impact of high blood sugar, insulin resistance is also an essential factor leading to the dysfunction of BMSCs. Under normal circumstances, insulin acts as a critical metabolic regulatory hormone that controls blood sugar levels and promotes cell proliferation and osteogenic differentiation through the PI3K/Akt signaling pathway (24). However, in patients with T2DM, insulin signal transduction pathways are inhibited, resulting in reduced activity of the PI3K/Akt signaling pathway, thereby affecting the osteogenic differentiation ability of BMSCs (4). Studies have shown that insulin resistance not only directly affects the function of BMSCs but also indirectly inhibits osteogenic differentiation by altering the levels of cytokines and growth factors in the microenvironment. For instance, insulin-like growth factor 1 (IGF-1) expression decreases in insulin-resistant states, and IGF-1 is an essential regulatory factor for BMSC osteogenic differentiation (12).

Multiple genes and signaling pathways play a crucial role in the osteogenic differentiation process of BMSCs. Among them, the Wnt/β-catenin signaling pathway is one of the fundamental regulatory mechanisms for osteogenic differentiation (10). Studies have shown that in patients with T2DM, the activity of the Wnt/β-catenin signaling pathway is significantly reduced, possibly due to the inhibitory effects of high blood sugar and insulin resistance on this pathway (9). Additionally, T2DM affects other osteogenic-related signaling pathways, such as the BMP/Smad and Notch signaling pathways (26). The dysregulation of these signaling pathways collectively leads to a decrease in the osteogenic capacity of BMSCs, further exacerbating the development of osteoporosis (3). Researchers have utilized various experimental methods and models to explore the osteogenic differentiation impairment of BMSCs in patients with T2DM. For example, simulating the metabolic status of T2DM patients by culturing BMSCs *in vitro* and exposing them to a high-sugar environment can observe the effects of high sugar on the function of BMSCs (2). Additionally, animal models such as T2DM mouse models allow the study of the behavior of BMSCs and changes in bone metabolism *in vivo* (25). These studies not only reveal the inhibitory effects of T2DM on the osteogenic differentiation of BMSCs but also provide a theoretical basis for finding therapeutic strategies to improve the function of BMSCs (4).

**Figure 2:**
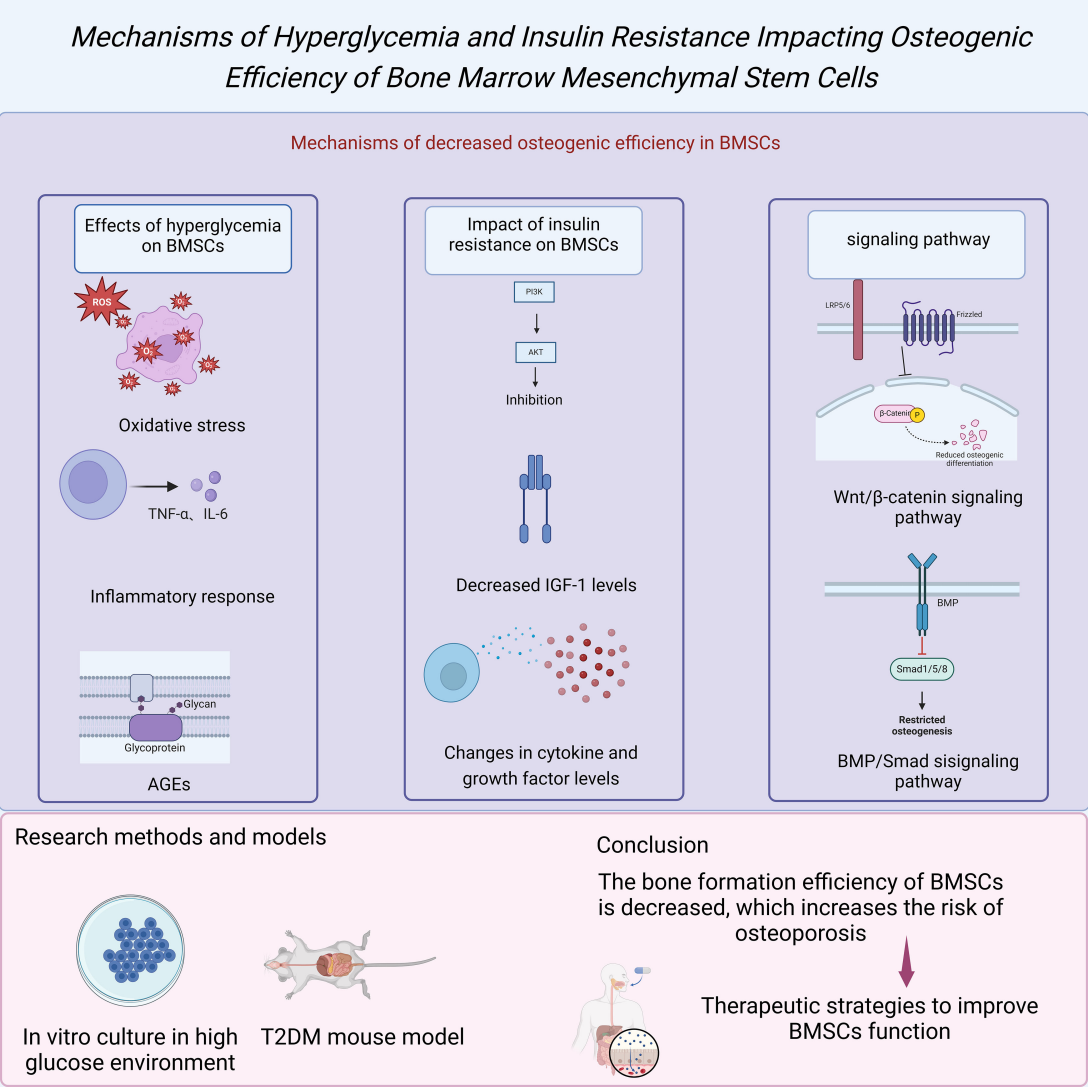
Mechanisms of hyperglycemia and insulin resistance impacting osteogenic efficiency of BMSCs.

In summary, the crucial role of BMSCs in bone formation makes them an essential target for research on T2DM-related osteoporosis. High blood sugar and insulin resistance inhibit the osteogenic differentiation capacity of BMSCs through multiple mechanisms, leading to a decrease in bone formation efficiency and an increased risk of osteoporosis (1). The implantation of BMSCs leads to weight gain in individuals with T2DM, significantly improves HFD-induced steatosis, and restores liver function as well as glucose and lipid metabolism disorders (27), offering a potential treatment for T2DM-related osteoporosis through BMSC implantation. Further research into these mechanisms not only aids in understanding the association between T2DM and osteoporosis and offers the potential for developing new treatment strategies (**Figure 2**). Future studies should continue to explore how to enhance the bone health of T2DM patients and reduce their disease burden by regulating the osteogenic differentiation of BMSCs.

## 4 Ferroptosis in bone cells

### 4.1 Overview of ferroptosis

Ferroptosis is a distinct form of cell death characterized by iron-dependent lipid peroxidation and oxidative stress. Iron, an essential trace element in the body, is critical in various physiological processes, including oxygen transport, DNA synthesis, and electron transfer. However, excess iron accumulation can lead to oxidative stress, causing cell damage and death (4). Studies have shown that ferroptosis is essential in various pathological conditions such as neurodegenerative diseases, cardiovascular diseases, and cancer (5). In the process of bone metabolism, the impact of ferroptosis on bone cells is particularly significant. Bone cells are the main components of bone tissue, including osteoblasts, osteoclasts, and osteocytes, which play crucial roles in bone remodeling and maintenance (3). Ferroptosis not only affects the survival of bone cells but also participates in the occurrence and development of osteoporosis by regulating intercellular signal transduction and gene expression.

### 4.2 The relationship between high blood sugar levels and ferroptosis

In patients with T2DM, high blood sugar levels can increase iron accumulation in the body, leading to ferroptosis induction. Under high blood sugar conditions, oxidative stress levels significantly increase in the body, impair the function of bone cells, and exacerbate osteoporosis by promoting inflammatory reactions (5). Studies have found that iron-regulatory protein expression is abnormal in bone cells of T2DM patients, leading to imbalanced iron metabolism (10). Specifically, in T2DM patients, there is an increase in the expression of Ferritin and Transferrin receptors, while the expression of the iron export protein Ferroportin is decreased, collectively resulting in iron accumulation in bone cells (4). The occurrence of ferroptosis depends on the presence of intracellular iron and ROS. High iron and ROS levels can trigger cell membrane damage and death through lipid peroxidation and glutathione depletion (6).

Ferroptosis impacts bone metabolism by directly affecting bone cells and exacerbates osteoporosis by influencing intercellular signal transduction and gene expression. Studies have shown that ferroptosis can activate various inflammatory signaling pathways, such as NF-κB and Mitogen-activated protein kinase (MAPK) pathways, which play a crucial role in the occurrence and progression of osteoporosis (2). Additionally, ferroptosis can regulate the function of osteoblasts and osteoclasts, affecting the bone remodeling process. Osteoblasts are responsible for bone formation, and ferroptosis inhibits their differentiation and mineralization capabilities, leading to decreased new bone formation (3). Osteoclasts are responsible for bone resorption, and ferroptosis influences the bone resorption process by regulating the activity and lifespan of osteoclasts (9).

### 4.3 Research progress on ferroptosis inhibitors

Researchers have explored various intervention strategies to counteract the damage of ferroptosis to bone cells. Iron chelators are a category of drugs that can bind to iron ions and promote their excretion, and have been shown to have potential in the prevention and treatment of ferroptosis-related diseases (25). For example, Deferoxamine is a commonly used iron chelator; studies have shown that it can significantly reduce iron accumulation and oxidative stress in bone cells of T2DM patients, improving osteoporosis conditions (5). In addition, antioxidants are also an important intervention strategy. By clearing excess ROS in the body, antioxidants can reduce lipid peroxidation and cell damage caused by ferroptosis (10). For example, N-acetylcysteine (NAC) as an effective antioxidant has been widely used in research and treatment of ferroptosis-related diseases (4).

**Figure 3:**
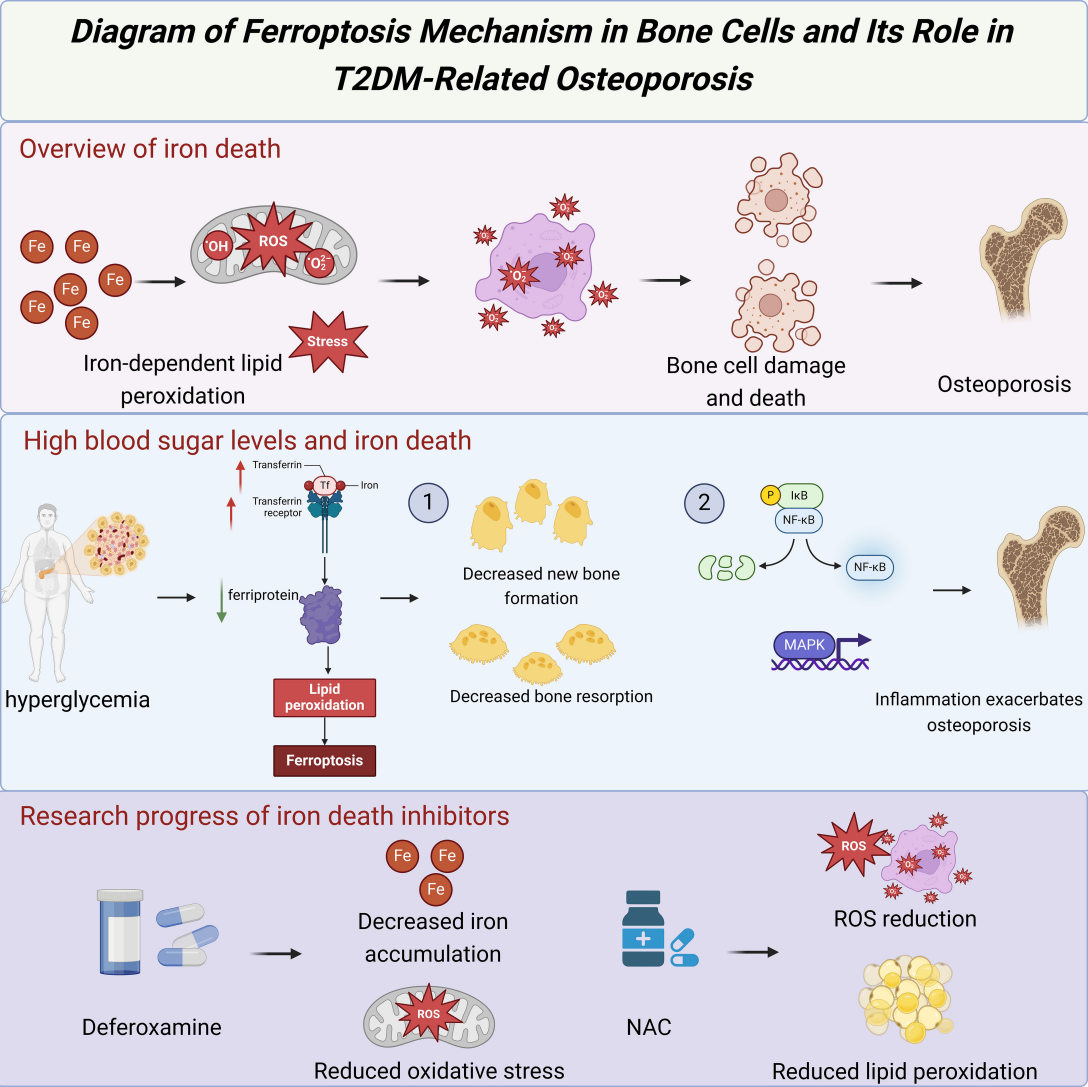
Diagram of ferroptosis mechanism in none cells and its role in T2DM-related osteoporosis.

Future research should further explore the specific mechanisms and potential therapeutic targets of ferroptosis in T2DM-related osteoporosis (**Figure 3**). Although several studies have already revealed the significant role of ferroptosis in the bone cells of T2DM patients, the exact molecular mechanisms are still unclear (6). For example, further investigation is needed to understand how ferroptosis affects bone cell function by regulating cell signaling pathways and gene expression. Additionally, the effectiveness and safety of intervention strategies such as iron chelators and antioxidants in clinical applications need more validation through clinical trials (2). By delving into the mechanisms of ferroptosis in T2DM-related osteoporosis, new treatment strategies could be developed to improve bone health and quality of life in T2DM patients (3).

## 5 Autophagy and osteoporosis

Autophagy is a critical intracellular degradation and recycling mechanism that maintains cellular homeostasis and function by degrading damaged organelles, proteins, and other cellular components through lysosomes (17). This process is equally important in bone cells, especially in response to oxidative stress and nutrient deficiency (18). In T2DM patients, the metabolic stress and inflammatory responses induced by hyperglycemia increase the risk of bone cell damage. Studies have shown that autophagy levels in the bone cells of T2DM patients are significantly elevated, representing a protective response to the stressful environment (16). However, prolonged hyperglycemia can lead to dysfunctional autophagy, resulting in abnormal expression of autophagy-related proteins and subsequently affecting bone health (20).

### 5.1 The role of mitophagy in T2DM

Mitophagy is a specialized form of autophagy responsible for clearing damaged or excess mitochondria to maintain mitochondrial quality and function (19). Mitochondrial dysfunction is a common pathological feature in patients with T2DM. The high glucose environment can lead to mitochondrial membrane depolarization and elevated levels of ROS, further damaging mitochondria and triggering mitophagy (20). Studies have found significantly increased levels of mitophagy in bone cells of patients with T2DM, which may serve as a compensatory mechanism to clear damaged mitochondria and reduce ROS accumulation (21). However, prolonged exposure to high glucose environments can disrupt mitophagy, exacerbating bone cell dysfunction and the progression of osteoporosis (22). NIPA2 is a protein associated with autophagy regulation, and studies have shown that it plays a crucial role in the bone cells of patients with T2DM (16). Under high glucose conditions, the expression of NIPA2 is significantly downregulated, leading to aberrantly elevated levels of mitophagy, thereby affecting the function and survival of bone cells (19). Furthermore, NIPA2 regulates mitophagy through the PGC-1α/FoxO3a pathway, which plays a crucial role in maintaining bone cell energy metabolism and antioxidative stress responses (17). Research has demonstrated that overexpression of NIPA2 can restore impaired mitophagy function under high glucose conditions, thereby improving the health of bone cells (18). These findings provide new potential targets and strategies for treating osteoporosis associated with T2DM.

### 5.2 The relationship between autophagy and bone

Autophagy in bone cell apoptosis and osteoporosis has also been widely studied. Under high glucose conditions, excessive autophagy can increase bone cell apoptosis, affecting bone health (20). Studies have shown that reducing the expression of autophagy-related proteins can decrease bone cell apoptosis and alleviate osteoporosis in patients with T2DM (19). Additionally, autophagy is closely associated with inflammatory responses, and excessive inflammation can exacerbate the progression of osteoporosis (21). Therefore, regulating the autophagy process and inflammatory responses may be an effective strategy for treating osteoporosis associated with T2DM. Autophagy plays a complex and vital role in the occurrence and development of osteoporosis in patients with T2DM. Autophagy is a cellular protective mechanism that helps maintain bone cell function and health under high glucose conditions by regulating mitophagy and coping with oxidative stress (17). However, sustained high glucose environments can lead to dysfunctional autophagy, further exacerbating bone cell damage and apoptosis (18). Through in-depth research on autophagy-related mechanisms and regulatory pathways, we can develop new treatment strategies to improve bone health in patients with T2DM, reduce the risk of fractures, and enhance quality of life (16). As a GLP-1 analog, Liraglutide has been shown to have an effective regulatory role in autophagy for treating diabetes (28). Exendin-4, a GLP-1 receptor antagonist, has also been found to reduce autophagic burden by promoting autophagosome clearance, thereby preventing Tac-induced islet damage (29).

**Figure 4:**
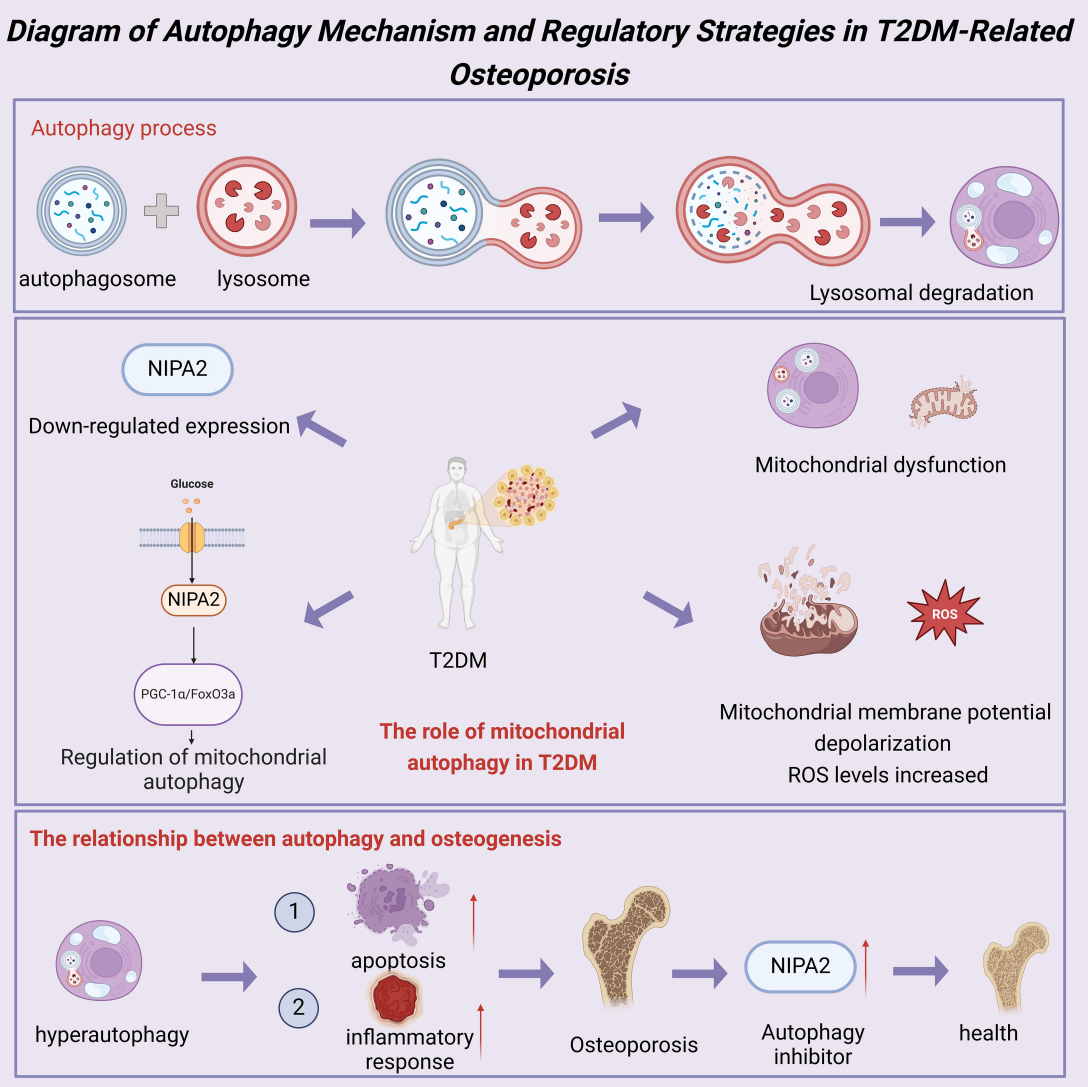
Diagram of autophagy mechanism and regulatory strategies in T2DM-related osteoporosis.

On the other hand, Metformin inhibits the proliferation of MIN6β cells and promotes apoptosis through AMPK-dependent and autophagy-mediated mechanisms (30). In addition to chemical drugs, traditional Chinese medicine compounds and extracts, such as Yunpi Heluo and Xiaokeping, have also been found to improve the progression of type 2 diabetes by modulating autophagy (31, 32). Future research should further explore the specific mechanisms of autophagy in osteoporosis associated with T2DM to provide a more solid scientific basis for clinical interventions (**Figure 4**).

## 6 Perirenal fat thickness and osteoporosis

**Figure 5:**
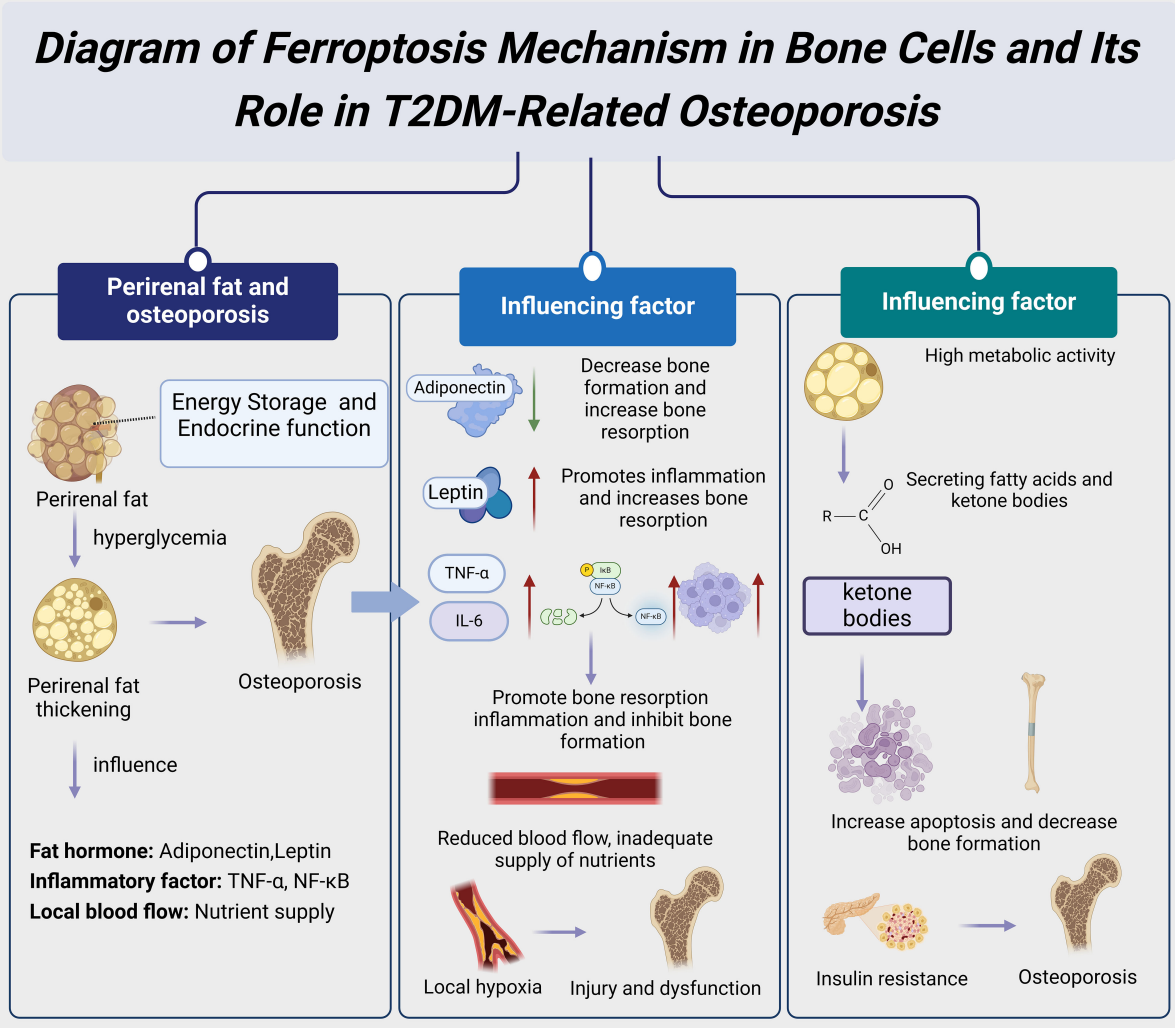
Diagram of the mechanisms and impacts of perirenal fat thickness in T2DM-related osteoporosis.

Perirenal fat refers to the fatty tissue surrounding the kidneys. Recent research indicates that this fat depot plays a significant role in energy storage, endocrine functions, and bone metabolism. The thickness of perirenal fat is closely associated with various metabolic diseases, particularly T2DM. T2DM patients often exhibit increased perirenal fat thickness, a change significantly linked to osteoporosis development and progression (13) (**Figure 5**). Perirenal fat influences bone metabolism through several mechanisms, including the secretion of adipokines and inflammatory factors and the alteration of local blood flow (14). These combined effects may exacerbate osteoporosis and increase the risk of fractures.

Adipokines secreted by perirenal fat play a crucial role in regulating bone metabolism. Adiponectin, a protein secreted by adipose tissue, is anti-inflammatory and enhances insulin sensitivity. Studies have found that adiponectin levels are often reduced in T2DM patients, which may lead to decreased bone formation and increased bone resorption, thereby promoting the development of osteoporosis (8). Additionally, perirenal fat secretes leptin, which regulates bone formation and resorption by binding to receptors on BMSCs. However, high leptin levels are negatively correlated with osteoporosis, as leptin’s pro-inflammatory effects may increase bone resorption (15). These findings suggest that adipokines secreted by perirenal fat have a complex regulatory role in T2DM-related osteoporosis. Perirenal fat also affects bone metabolism by secreting various inflammatory factors. In a hyperglycemic state, the perirenal adipose tissue of T2DM patients secretes significant amounts of inflammatory cytokines such as TNF-α and IL-6 (10). These inflammatory cytokines activate the NF-κB signaling pathway, promoting bone resorption and inhibiting bone formation, thus contributing to the development and progression of osteoporosis (9). Additionally, the increased number of macrophages in perirenal fat can exacerbate the inflammatory response, further affecting bone health (11). Therefore, controlling the inflammatory response in perirenal fat may be an effective strategy for treating T2DM-related osteoporosis.

Perirenal fat thickness may further influence bone metabolism by affecting local blood flow and nutrient supply. Studies indicate that increased perirenal fat thickness can compress local blood vessels, reducing blood flow to the kidneys and surrounding bone tissues (14). This reduction in blood flow affects the nutrient supply to bone tissue and may cause local hypoxia, exacerbating bone cell damage and dysfunction (15). Additionally, the increased perirenal fat thickness may alter the local metabolic environment, affecting the bone tissue’s response to hormones and growth factors, thereby impacting bone metabolism (13). Thus, the increase in perirenal fat thickness may be a significant risk factor for the development of osteoporosis in T2DM patients. Furthermore, the high metabolic activity of perirenal fat may affect bone metabolism through other mechanisms. For instance, studies have found that adipocytes in perirenal fat have high metabolic activity and can secrete various bioactive substances, such as fatty acids and ketone bodies (14). At high concentrations, these substances have toxic effects on bone cells, potentially leading to increased apoptosis and decreased bone formation (13). Moreover, perirenal fat may indirectly affect bone metabolism by influencing systemic metabolic states, such as increasing insulin resistance and hyperglycemia (10). Therefore, understanding the metabolic characteristics of perirenal fat and its impact on bone metabolism is crucial for elucidating the mechanisms of T2DM-related osteoporosis.

## 7 VDR gene polymorphism

**Figure 6:**
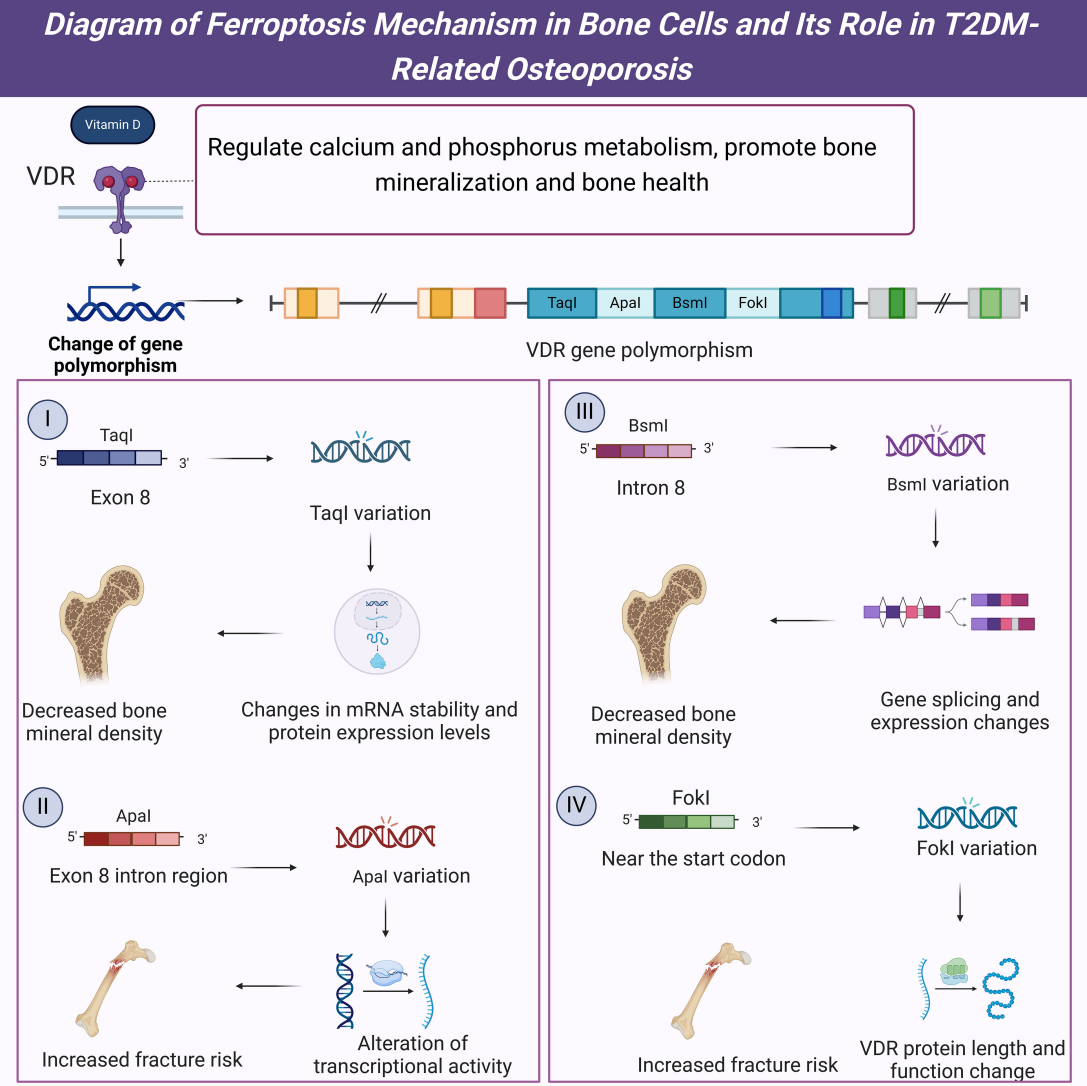
Diagram of the mechanisms and impacts of VDR gene polymorphisms in T2DM-related osteoporosis.

Vitamin D is crucial in bone health, primarily through the VDR. VDR is a nuclear receptor widely expressed in various tissues and cells, including bone cells, intestinal epithelial cells, and immune cells. Vitamin D binds to VDR to regulate calcium and phosphorus metabolism, promoting bone mineralization and overall bone health (26). Studies have shown that polymorphisms in the VDR gene significantly affect an individual’s susceptibility to osteoporosis (25) (**Figure 6**). These genetic polymorphisms may alter VDR expression or function, thereby influencing the bioactivity of vitamin D and its role in bone metabolism (1). In T2DM patients, VDR gene polymorphisms may further increase the risk of osteoporosis, a phenomenon that has garnered considerable attention.

Firstly, the TaqI polymorphism (rs731236) in the VDR gene is one of the most extensively studied genetic variations. This polymorphism in exon 8 of the VDR gene affects the stability of VDR mRNA and the expression levels of the VDR protein (18). Studies have shown that individuals carrying the TaqI polymorphism have significantly lower bone mineral density (BMD) compared to those without this variation, and this phenomenon is more pronounced in T2DM patients (17). Furthermore, the TaqI polymorphism is closely associated with serum vitamin D levels, with carriers having lower levels of vitamin D, which may further impact their bone health (20). These findings suggest that the TaqI polymorphism is crucial in developing T2DM-related osteoporosis. Secondly, the ApaI polymorphism (rs7975232) in the VDR gene is also associated with osteoporosis susceptibility. The ApaI polymorphism, located in an intronic region of exon 8, may influence the transcriptional activity of the VDR gene (19). Research indicates that individuals carrying the ApaI polymorphism have significantly lower BMD than those without this variation, which is more pronounced in T2DM patients (17). Additionally, the ApaI polymorphism is associated with an increased risk of fractures, with carriers having a significantly higher fracture risk than non-carriers (18). These results indicate that the ApaI polymorphism plays an important role in the occurrence and development of T2DM-related osteoporosis and may serve as a potential diagnostic marker for T2DM-related osteoporosis.

The BsmI polymorphism (rs1544410) in the VDR gene is another common genetic variation. Located in intron 8 of the VDR gene, the BsmI polymorphism may influence the splicing and expression of the VDR gene (19). Studies have shown that individuals carrying the BsmI polymorphism have significantly lower BMD than those without this variation, a notably pronounced phenomenon in T2DM patients (17). Additionally, the BsmI polymorphism is closely associated with serum vitamin D levels and bone turnover markers, with carriers exhibiting lower vitamin D levels and higher bone turnover marker levels, which may further impact their bone health (20). These findings suggest that the BsmI polymorphism has an important regulatory role in T2DM-related osteoporosis and may serve as a potential diagnostic marker for T2DM-related osteoporosis. Furthermore, the FokI polymorphism (rs2228570) is located near the start codon of the VDR gene and directly affects the length and function of the VDR protein (18). Research has shown that individuals carrying the FokI polymorphism have significantly lower BMD than those without this variation, with this effect being more pronounced in T2DM patients (17). Additionally, the FokI polymorphism is associated with an increased risk of fractures, with carriers having a significantly higher fracture risk than non-carriers (19). These results suggest that the FokI polymorphism plays a vital role in the development and progression of T2DM-related osteoporosis and may also serve as a potential diagnostic marker for T2DM-related osteoporosis.

## 8 Mitochondrial defects and osteoporosis

**Figure 7:**
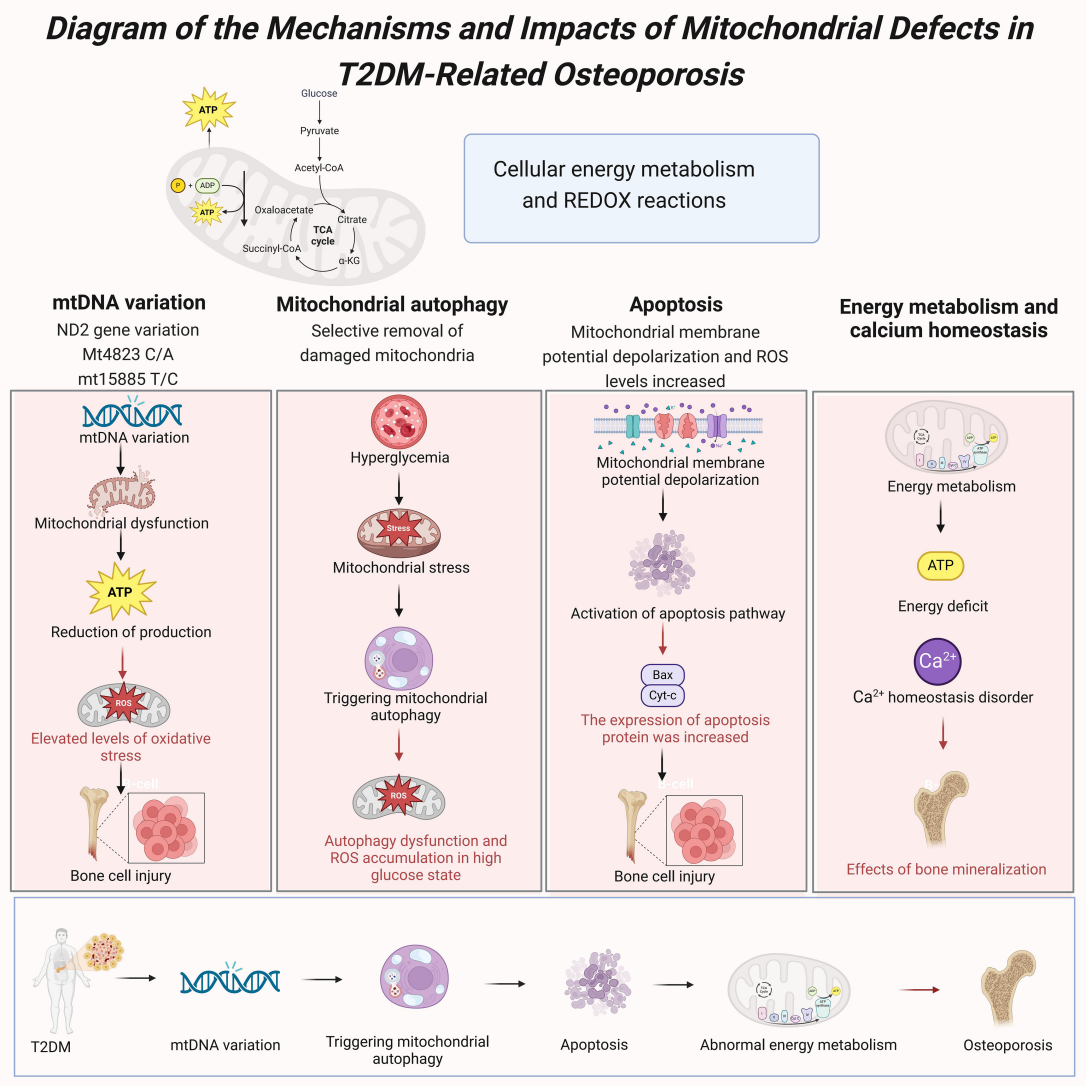
Diagram of the mechanisms and impacts of mitochondrial defects in T2DM-related osteoporosis.

Mitochondria play a crucial role in cellular energy metabolism and redox reactions, and their proper functioning is essential for maintaining cellular health and function. In bone cells, mitochondrial dysfunction is considered a significant factor in the onset and progression of osteoporosis. The hyperglycemic environment commonly found in T2DM patients induces mitochondrial stress responses, impairing mitochondrial function and subsequently affecting bone metabolism (33). Studies have shown that mitochondrial defects are significantly associated with the high prevalence of osteoporosis in T2DM patients, offering new insights into the mechanisms linking these two conditions (34) (**Figure 7**).

Mitochondrial DNA (mtDNA) variations play a crucial role in T2DM-related osteoporosis. Mutations in the mitochondrial genome can lead to mitochondrial dysfunction, affecting ATP production and the oxidative phosphorylation process (33). Studies have found that the mutation rate of mtDNA in bone cells of T2DM patients is significantly higher than in healthy individuals, and these mutations are closely associated with decreased mitochondrial function and the development of osteoporosis (35). Specifically, NADH dehydrogenase 2 gene (ND2) variations can increase oxidative stress levels, exacerbating bone cell damage and dysfunction (34). Studies have also identified specific mtDNA variations, such as Mt4823 C/A and mt15885 T/C, that are significantly associated with the development of osteoporosis in T2DM patients (33). Mitophagy plays a critical role in maintaining mitochondrial function and cellular health by selectively removing damaged or excess mitochondria, thus helping to maintain the balance of mitochondrial quality and quantity (20). In T2DM patients, the hyperglycemic environment enhances mitochondrial stress responses, triggering mitophagy (19). However, prolonged hyperglycemia can lead to dysfunctional mitophagy, which fails to effectively clear damaged mitochondria, accumulating ROS and further deteriorating mitochondrial function (20). Research indicates that abnormal levels of mitophagy in the bone cells of T2DM patients are closely related to the onset and progression of osteoporosis (17).

Mitochondrial dysfunction exacerbates the development of osteoporosis by influencing apoptosis pathways. In a hyperglycemic environment, mitochondrial membrane potential depolarization and elevated ROS levels induce apoptosis in bone cells (20). Studies have shown that mitochondrial-related apoptosis pathways are significantly active in the bone cells of T2DM patients, likely due to mitochondrial stress responses triggered by high glucose levels (19). Additionally, the expression levels of mitochondrial apoptotic proteins such as Bax and cytochrome c (Cyt-c) are significantly increased in T2DM patients. These proteins mediate apoptosis through mitochondrial pathways, leading to increased bone cell loss and the progression of osteoporosis (17). The critical role of mitochondria in energy metabolism directly impacts the function and health of bone cells. In T2DM patients, mitochondrial dysfunction in bone cells leads to reduced ATP production, resulting in insufficient energy supply (33). This energy metabolism abnormality not only impairs bone cells’ normal function but also decreases osteoblasts’ activity, reducing new bone formation (34). Furthermore, mitochondrial dysfunction disrupts calcium ion homeostasis, which is crucial for bone mineralization (20). Studies indicate that mitochondrial dysfunction in the bone cells of T2DM patients is closely associated with the development of osteoporosis, involving mechanisms related to energy metabolism, calcium ion homeostasis, and apoptosis (19).

In summary, mitochondrial defects are crucial in the onset and progression of T2DM-related osteoporosis. Key factors contributing to osteoporosis in T2DM patients include mtDNA variations, dysregulated mitophagy, mitochondrial dysfunction, and the resulting apoptosis and energy metabolism abnormalities (17, 33, 34).

## 9 Individualized treatment for T2DM

Personalized treatment is crucial in managing T2DM and osteoporosis due to the variations in patients’ age, gender, medical history, genetic polymorphisms, and lifestyle factors. Treatment plans need to be tailored to individual circumstances. Age is a crucial factor influencing treatment strategies. Older patients face a higher risk of fractures and multiple complications, necessitating a greater focus on maintaining bone density and implementing fall prevention measures (1). Younger patients may be more concerned with long-term bone health management to prevent early-onset osteoporosis. Gender differences also play a significant role. Postmenopausal women are more susceptible to osteoporosis due to decreased estrogen levels, requiring special attention to hormone replacement therapy and vitamin D supplementation (25). Male patients should focus on lifestyle interventions such as increasing physical activity and managing weight. Additionally, a patient’s medical history, including the duration and control of diabetes, history of fractures, and presence of other chronic diseases, should be considered in the personalized treatment plan.

Additionally, clinical studies have shown that the anti-osteoporosis drug Alendronate demonstrates comparable vertebral anti-fracture efficacy in both diabetic and non-diabetic patients (n=2), and Raloxifene also shows similar vertebral anti-fracture efficacy between the two groups (n=2). Teriparatide (n=1) has exhibited the same non-vertebral fracture rate in patients with and without type 2 diabetes (36). Thus, diabetes does not alter the response to anti-osteoporosis treatment, indicating the need to appropriately incorporate anti-osteoporosis medications based on the patient’s condition in personalized therapy. More effective treatment plans can be developed by comprehensively evaluating these factors, ultimately improving treatment outcomes and patient quality of life.

### 9.1 Development of an individualized treatment plan

Developing personalized treatment plans requires detailed patient assessment and precise medical data. Firstly, clinicians should conduct comprehensive medical history inquiries and physical examinations to understand the patient’s baseline condition and specific needs. Secondly, genetic testing can provide crucial information, particularly the detection of VDR gene polymorphisms (such as TaqI and EcoRV), which are significant for predicting osteoporosis risk and guiding vitamin D supplementation (26). Furthermore, imaging examinations like dual-energy X-ray absorptiometry (DXA) can accurately assess bone density and help determine the severity of osteoporosis. Combining this with biochemical indicators, such as blood glucose levels, calcium-phosphorus metabolism, and bone turnover markers, can offer a more comprehensive understanding of the patient’s bone health. Based on these assessments, physicians can formulate individualized treatment plans. For example, in patients with severe osteoporosis, bisphosphonates or other anti-resorptive drugs can be considered. An intermittent PTH administration strategy may significantly promote bone formation and reduce fracture risk (37). Additionally, a strategy involving selective estrogen receptor modulators (SERMs), such as Raloxifene (Evista), can also be considered (38), along with the reinforcement of calcium and vitamin D supplementation. For patients with concurrent obesity, weight reduction and increased physical activity are also essential components of treatment. Developing personalized treatment plans should focus on disease management and consider the patient’s quality of life and long-term health management.

### 9.2 Future development direction of individualized treatment

Significant progress has been made in personalized treatment for T2DM and osteoporosis, yet many challenges remain. Current research primarily focuses on the individualized selection of pharmacotherapy. For instance, studies have found that certain bisphosphonates are more effective in specific genetic backgrounds (3). Additionally, genetic testing advancements have enabled gene polymorphisms to be applied in personalized treatment. Research indicates that VDR gene polymorphism is closely related to the risk of osteoporosis, providing a theoretical basis for gene-based personalized therapy. However, translating these research findings into clinical practice requires further exploration. Personalized treatment is mainly limited to large medical centers and research institutions, with dissemination and implementation in primary care settings facing significant challenges. Moreover, patient adherence and long-term management are critical factors that influence treatment outcomes. Future research should focus on the practicality and feasibility of personalized treatment plans, exploring new therapeutic targets and methods to enhance treatment efficacy and improve patient quality of life.

In the future, personalized treatment will play an increasingly significant role in managing T2DM and osteoporosis. With the advancement of precision medicine, genetic testing and biomarkers will become more widespread, providing more evidence and tools for personalized therapy. Artificial intelligence and big data technologies will also greatly enhance the efficiency and accuracy of developing personalized treatment plans. For example, by analyzing large datasets of patient information, predictive models can be established to help physicians formulate more precise treatment strategies (17). Future research should emphasize interdisciplinary collaboration, integrating knowledge from clinical medicine, genetics, nutrition, and sports medicine to offer patients more comprehensive and personalized health management plans. Patient education and health management should also be integral components of personalized treatment, helping patients understand their condition and treatment plans, thereby improving adherence and self-management capabilities. Overall, the future direction should focus on improving treatment outcomes and patient quality of life, advancing personalized treatment research and application, and providing more precise and effective medical services.

## 10 Future prospects and research

Future research on the mechanisms linking T2DM and osteoporosis should deeply explore the influence of gene-environment interactions on osteoporosis susceptibility. Existing studies have identified several gene polymorphisms associated with osteoporosis, such as VDR gene polymorphisms, which affect individual responses to vitamin D and, consequently, bone density and health (26). Future research can utilize genome-wide association studies (GWAS) and epigenetic studies to further identify susceptibility genes related to T2DM-associated osteoporosis and investigate these genes’ expression and functional changes under different environmental factors (1). Additionally, an in-depth exploration of the regulatory mechanisms of vitamin D receptor (VDR) gene polymorphisms in T2DM-associated osteoporosis is warranted, including the specific signaling pathways these receptor genes participate in and their correlation with insulin or glucose metabolism. By employing gene editing technologies such as CRISPR-Cas9, the roles of these genes in T2DM-related osteoporosis can be validated in both *in vivo* and *in vitro* models (25). Studying gene-environment interactions will provide new perspectives on how these genes function in various metabolic states, thereby offering a basis for developing personalized treatment strategies.

The role of autophagy in T2DM-related osteoporosis is another crucial area for future research. As an essential cellular protective mechanism, autophagy exhibits abnormalities in a high-glucose environment, which may be a critical factor in the development of osteoporosis (17). Research should focus on autophagy-related proteins and their signaling pathways, such as mTOR, AMPK, and PI3K/Akt. By targeting these pathways, we can explore their specific roles in bone metabolism (19). The relationship between autophagy and mitochondrial function is also an important research direction. Mitophagy is vital in removing damaged mitochondria, maintaining cellular energy metabolism, and reducing oxidative stress. Its dysregulation may be a key mechanism in T2DM-related osteoporosis (20). By investigating the interaction between autophagy and mitochondrial function and the specific regulatory mechanisms of mitophagy in T2DM-related osteoporosis, we can gain a deeper understanding of their roles in bone metabolism and develop new therapeutic strategies to improve bone health in T2DM patients. Additionally, the potential and feasibility of various established autophagy-related drugs for clinical application in T2DM-associated osteoporosis represent key areas for future research focus in this field.

Interdisciplinary and international collaboration is crucial in studying the comorbid mechanisms of T2DM and osteoporosis. Research on T2DM and osteoporosis involves multiple fields, including endocrinology, bone metabolism, genetics, and molecular biology (1). Interdisciplinary collaboration allows for integrating research findings from various fields, providing a comprehensive understanding of the mechanisms linking T2DM and osteoporosis (25). For instance, combining clinical research with basic science can better explain clinical observations and translate laboratory discoveries into clinical applications (17). Additionally, international cooperation is vital for future research, as sharing research resources and data can accelerate the progress of studies on the mechanisms related to T2DM and osteoporosis (18). In summary, interdisciplinary and international collaboration can enhance the depth and breadth of research, offering more comprehensive treatment plans for T2DM patients and ultimately improving their quality of life and health outcomes.

## 11 Conclusion

**Figure 8:**
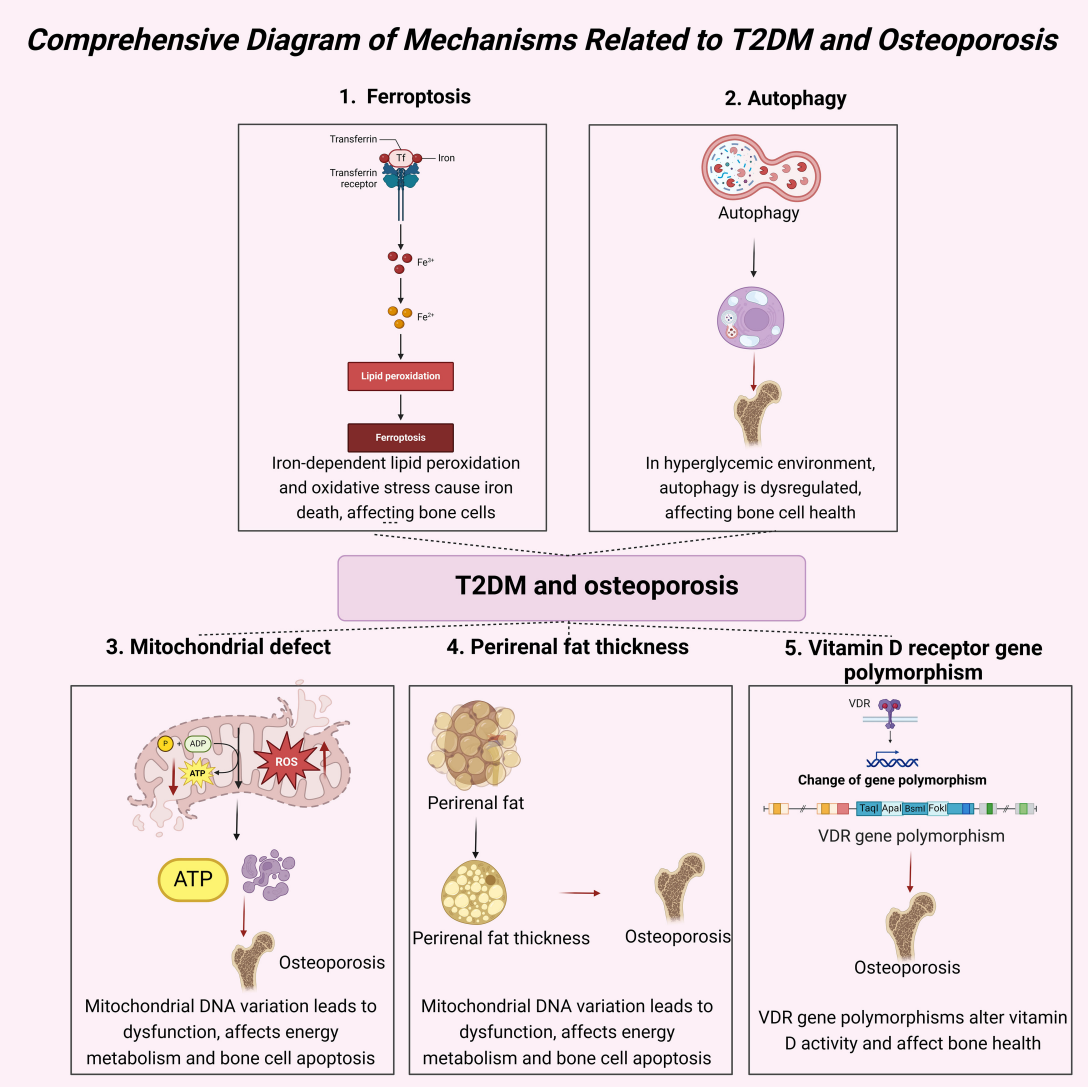
Comprehensive diagram of mechanisms related to T2DM and osteoporosis.

In summary, there are complex and multifaceted mechanisms linking T2DM and osteoporosis. These mechanisms include hyperglycemia-induced oxidative stress, chronic inflammation, insulin resistance, dysregulated autophagy, increased perirenal fat thickness, and mitochondrial dysfunction, among other biological pathways. **Table 1** provides a detailed summary of these mechanisms and their interrelationships, illustrating the complex connections between T2DM and osteoporosis. This table visually represents the interactions among decreased bone formation, ferroptosis, autophagy, perirenal fat thickness, VDR gene polymorphism, and their combined impact on osteoporosis. Hyperglycemia and insulin resistance are core pathological features of T2DM, directly affecting bone cell function and activating various cellular signaling pathways that promote bone resorption and inhibit bone formation, thereby increasing the risk of osteoporosis. Especially notable are oxidative stress and inflammatory responses, which are highly active in T2DM patients, leading to bone cell damage and apoptosis, thus exacerbating osteoporosis. Additionally, perirenal adipose tissue, functioning as an active endocrine organ, secretes adipokines and inflammatory factors that further disrupt bone metabolic balance, promoting the progression of osteoporosis. Mitochondrial dysfunction is common in T2DM patients, affecting energy metabolism and leading to cell apoptosis and increased oxidative stress, directly causing bone cell damage and functional impairment. These complex biological mechanisms contribute to the high prevalence of osteoporosis in T2DM patients. **Figure 8** provides an overview of these mechanisms, illustrating the roles of decreased bone formation, ferroptosis, autophagy, perirenal fat thickness, and VDR gene polymorphism in this process.

**Table 1:** Summary of the mechanisms related to T2DM and osteoporosis.

Mechanism	Specific function	Interrelationship
Decreased bone formation	The decreased bone formation capacity of BMSCs in patients with T2DM is associated with abnormal bone metabolism and reduced osteoblast activity.	Indirectly inhibiting bone formation through the influence of polymorphisms in the vitamin D receptor gene and perirenal fat thickness.
Ferroptosis	High blood glucose levels lead to increased ferroptosis of osteoblasts, particularly associated with oxidative stress and lipid peroxidation.	By increasing oxidative stress and exacerbating cellular damage, the decrease in bone formation is promoted. Disruption of mitochondrial function during autophagy processes also contributes to the occurrence of ferroptosis.
Autophagy	Abnormalities in mitochondrial autophagy are commonly present in patients with T2DM, affecting the function of osteoblasts.	Directly associated with ferroptosis, the combined action of mitochondrial autophagy and ferroptosis can lead to the death of osteoblasts, further inhibiting bone formation.
Perirenal fat thickness	The increase in perirenal fat thickness is associated with the development of osteoporosis, which is related to the secretion of adipokines and local inflammatory status.	By generating inflammatory factors and adipokines that impact bone formation, indirectly affecting ferroptosis and the autophagy process through pathways such as adiponectin.
Vitamin D receptor gene polymorphism	Polymorphisms in the vitamin D receptor gene (such as TaqI and EcoRV) impact the metabolism of vitamin D and bone metabolism, thereby influencing the development of osteoporosis.	Possibly indirectly influencing ferroptosis and the autophagy process through the secretion of adipokines, potentially by regulating the bone formation capacity of BMSCs and perirenal fat thickness.

Future research should continue to delve deeper into these mechanisms to develop more effective prevention and treatment strategies. Firstly, studying the interaction between genes and the environment will be crucial. Through GWAS and epigenetic research, more susceptibility genes related to osteoporosis can be identified, and the expression and functional changes of these genes under different environmental factors can be explored, providing new therapeutic targets. Secondly, research on autophagy and mitochondrial function will reveal more about the dysregulation of cellular protective mechanisms in high-glucose environments, laying the theoretical foundation for developing novel therapeutic strategies. In particular, studies on the regulation of mitophagy will help understand how to maintain bone cell health by clearing damaged mitochondria. Moreover, interdisciplinary and international collaboration will accelerate progress in this research field. Integrating research findings from different disciplines can provide a comprehensive understanding of the mechanisms linking T2DM and osteoporosis and translate laboratory discoveries into clinical applications, improving bone health and quality of life for T2DM patients. In conclusion, a deeper understanding of the complex pathological connections between T2DM and osteoporosis will offer new hope and directions for developing personalized treatment plans, reducing fracture risk, and enhancing patients’ quality of life.
